# Supplementary material for: Splicing QTL analysis focusing on coding sequences reveals mechanisms for disease susceptibility loci
Source: Nat Commun. 2022 Aug 24;13:4659. doi: 10.1038/s41467-022-32358-1 (PMC9402578; doi:10.1038/s41467-022-32358-1)
Supplement: Supplementary file 1 — Supplementary Information [file 41467_2022_32358_MOESM1_ESM.pdf]

Supplementary Figure 1. Accuracy of three isoform quantification methods evaluated using RNA-seq simulation data

a b

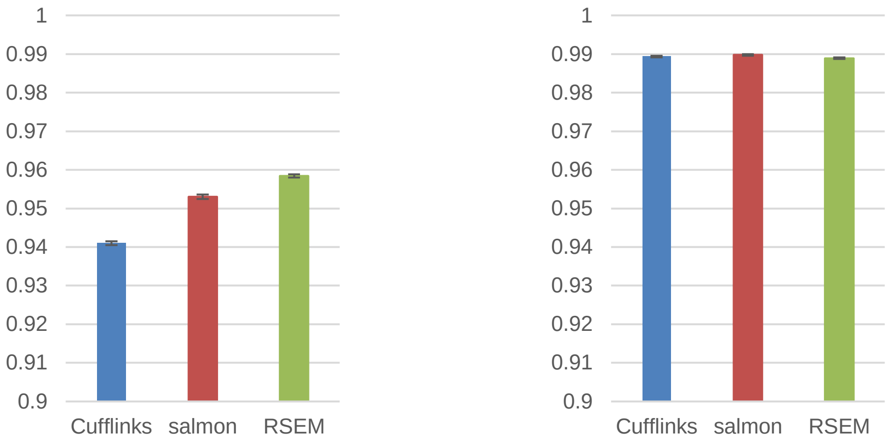

a. Mean of Spearman correlation coefficients between isoform FPKMs calculated using the three methods and the true isoform FPKMs in simulation data. The whiskers in the figure indicate 95% confidence intervals. The respective means are Cufflinks 0.9410, salmon 0.9530, and RSEM 0.9584 (n = 105). The error bars in the figure indicate standard errors.
b. Mean of Spearman correlation coefficients between isoform ratios calculated using the three methods and the true isoform ratios in simulation data. The whiskers in the figure indicate 95% confidence intervals. The respective means are Cufflinks 0.9894, salmon 0.9898, and RSEM 0.9889 (n = 105). The error bars in the figure indicate standard errors.

Supplementary Figure 2. Accuracy of i-rQTL analysis evaluated using simulation data

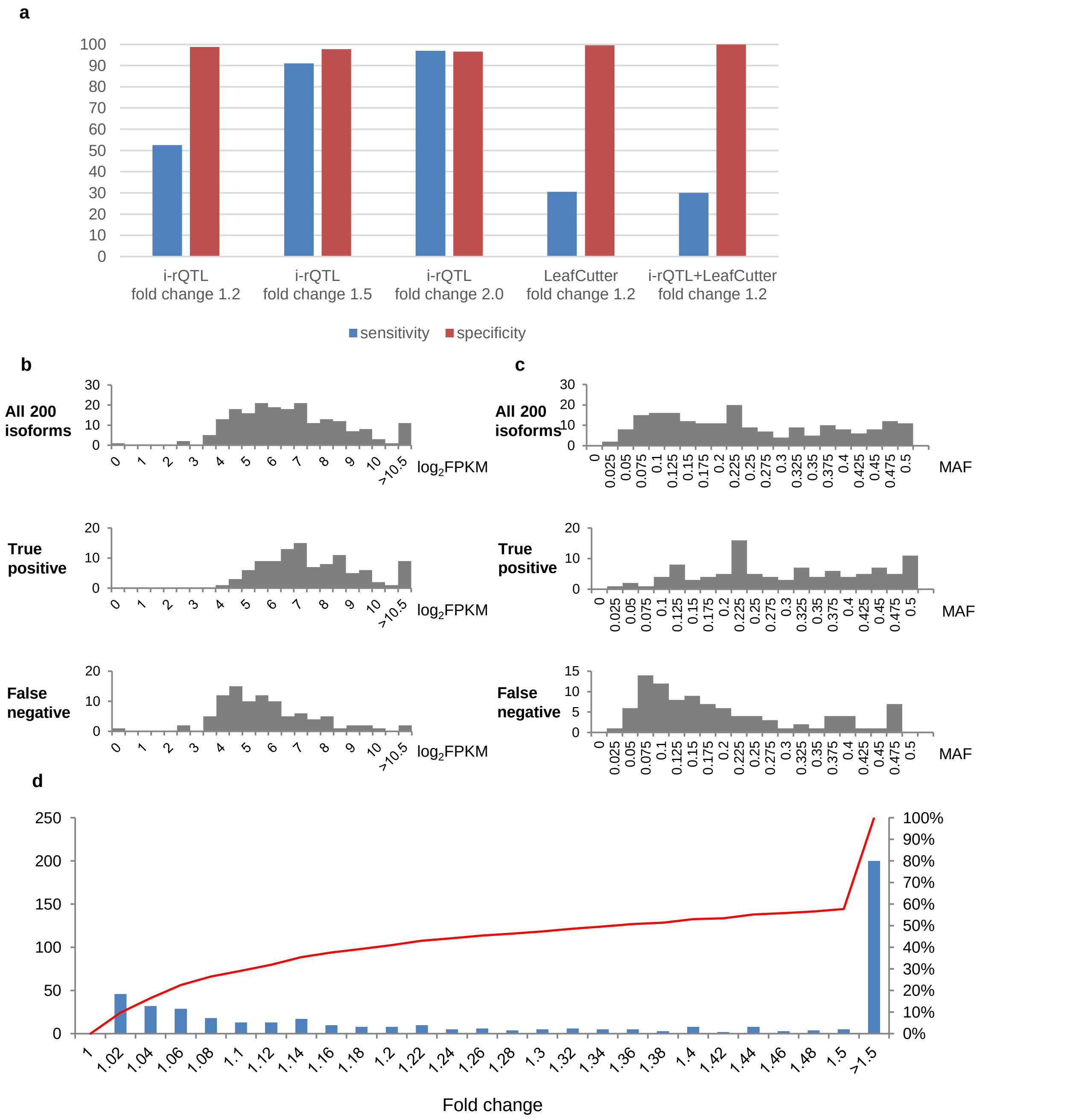

a. Sensitivity and specificity of i-rQTL analysis or LeafCutter or a combination of both analyses for each effect size.

b. Average of 200 differentially expressed isoforms, 105 true positive isoforms, and 95 false negative isoforms in the simulated i-rQTL analysis performed with a fold change of 1.2.

c. MAFs (minor allele frequencies) of simulated QTL variants corresponding to each classification in b.

d. Effect sizes of isoform eQTL obtained from analysis using real data. The red line indicates the cumulative percentage (right axis).

Supplementary Figure 3. Comparison of LeafCutter and i-rQTL analyses

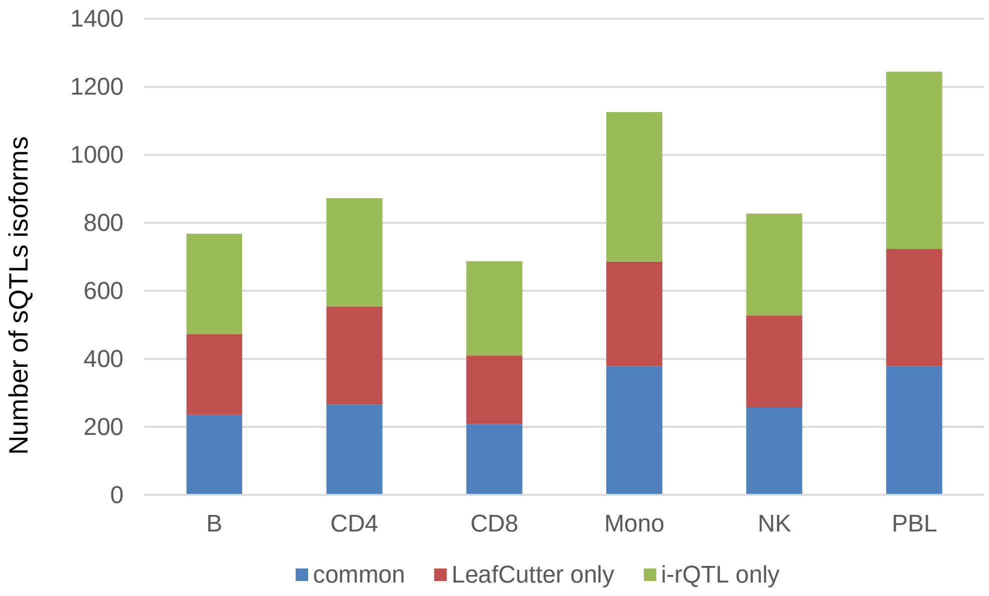

The number of significant sQTL isoforms identified in LeafCutter or in i-rQTL analysis or in both analyses. Only isoforms with a unique junction are shown (B cell 27,468, $CD4^+$ 27,784, $CD8^+$ 27,703, Monocyte 28,023, NK cell 29,841, PBL 30,330 isoforms).

Supplementary Figure 4. Evaluation of multimodality of lead i-rQTL distribution using silverman test

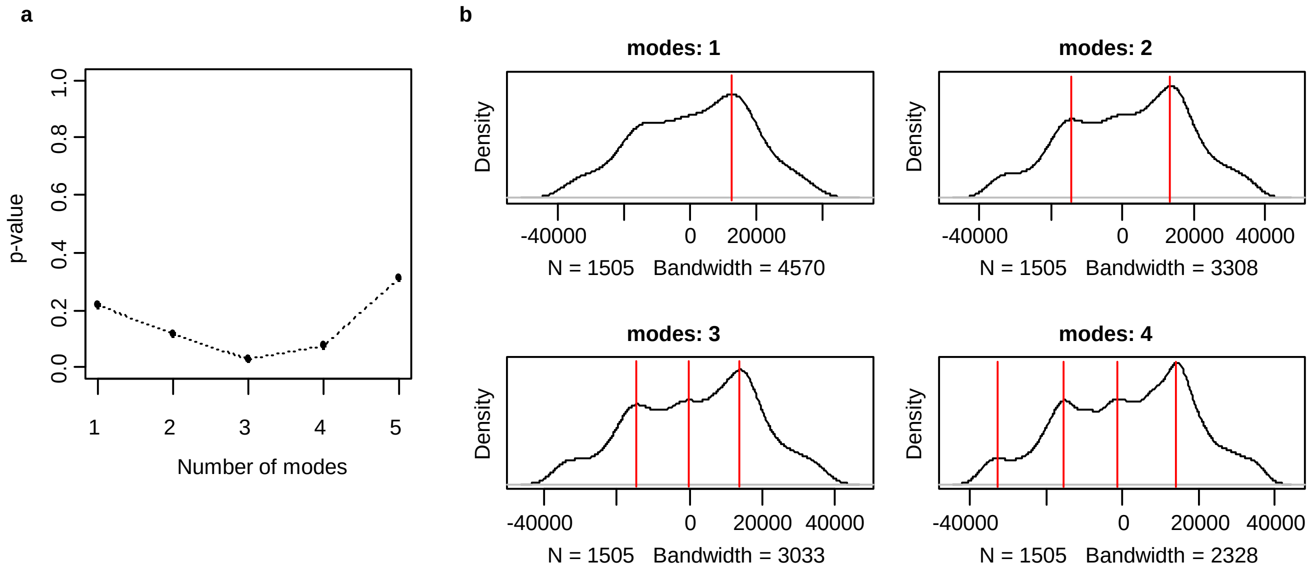

a. p-values of the silverman test in each mode performed on the distribution of i-rQTL variants in Figure 1e.
b. The position and bandwidth of the peaks for each mode. On the x-axis, TSS corresponds to -15,000 and TES to 15,000.

Supplementary Figure 5. Differential distribution of i-rQTL lead variants by cell type specificity using mash analysis

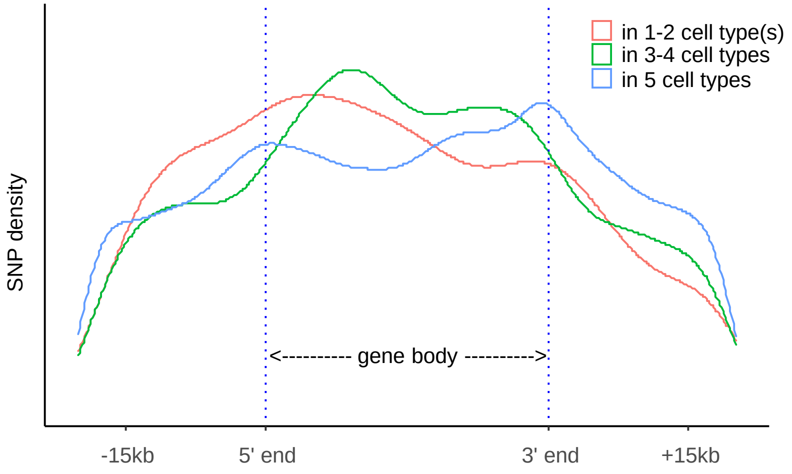

The results of i-rQTL analysis were meta-analyzed using mash (multivariate adaptive shrinkage) analysis to assess the cell-type specificity. As a result, 8735 of the 9094 i-rQTL genes had a common i-rQTL effect in all cell types, and only 13 i-rQTL genes had one cell-type specific i-rQTL effect. The distribution of lead i-rQTL variants relative to the gene body for each cell-type specificity were shown. The cell-type specificities were classified as in 1-2 cell type(s), 3-4 cell types, and 5 cell types to avoid extreme differences in the number of genes in each category.

Supplementary Figure 6. Full-length of CDSI isoforms identified by long-read capture RNA-seq

Major completed CDS incomplete isoforms account for more than half of the expression of isoforms with CDSI-specific junctions.

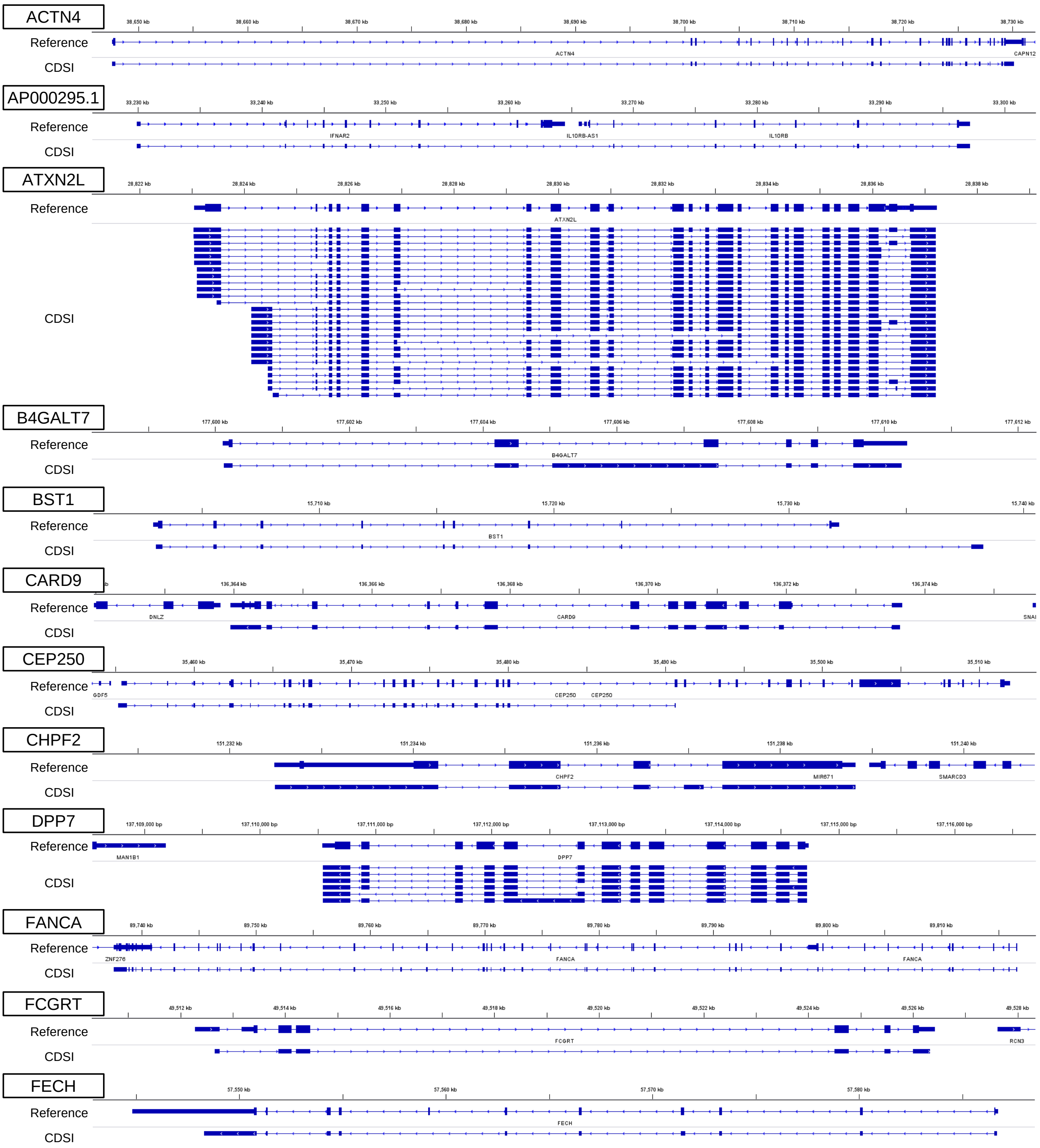

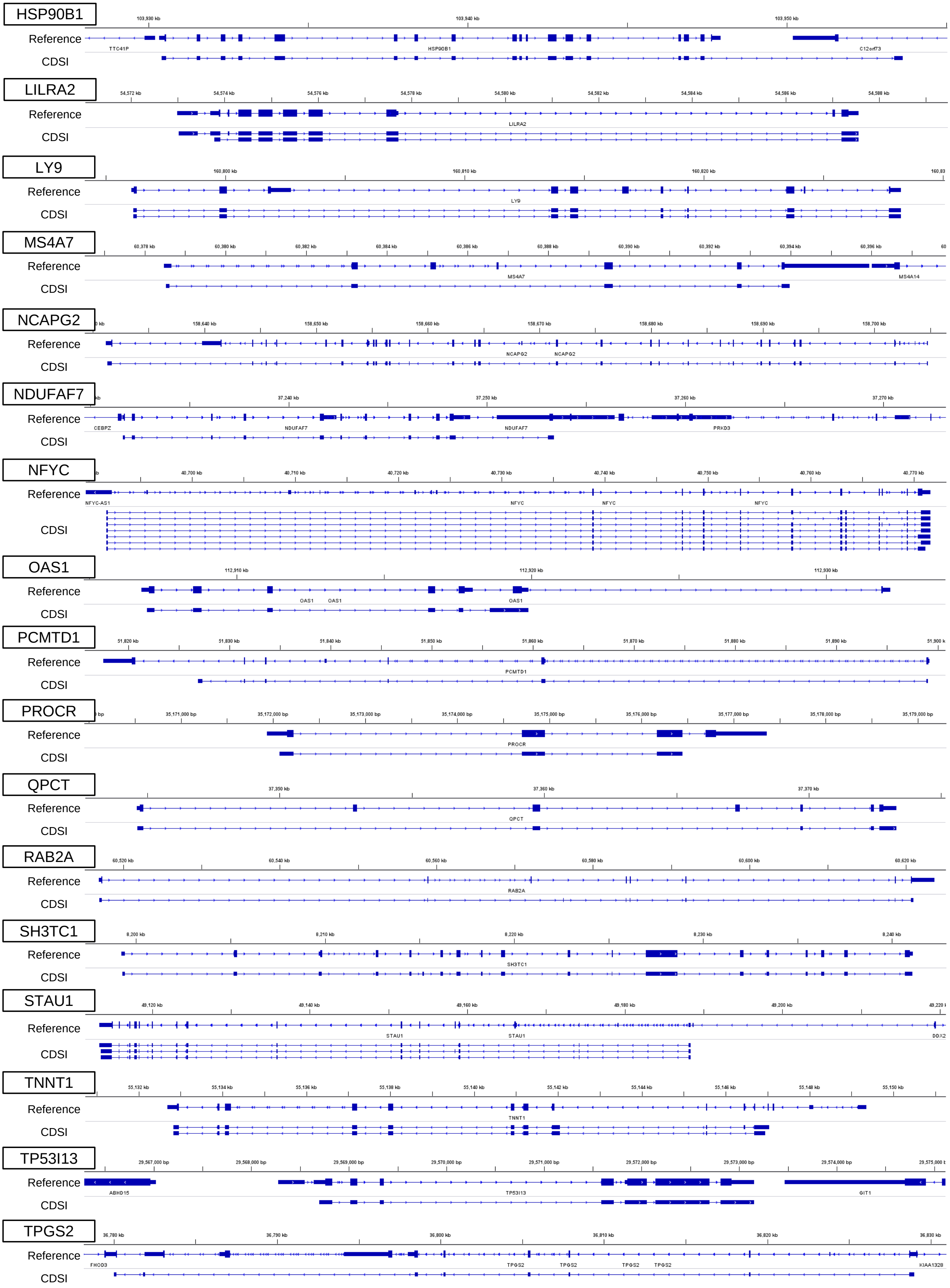
HSP90B1
Reference
CDSI
LILRA2
Reference
CDSI
LY9
Reference
CDSI
MS4A7
Reference
CDSI
NCAPG2
Reference
CDSI
NDUFAF7
Reference
CDSI
NFYC
Reference
CDSI
OAS1
Reference
CDSI
PCMTD1
Reference
CDSI
PROCR
Reference
CDSI
QPCT
Reference
CDSI
RAB2A
Reference
CDSI
SH3TC1
Reference
CDSI
STAU1
Reference
CDSI
TNNT1
Reference
CDSI
TP53I13
Reference
CDSI
TPGS2
Reference
CDSI

Supplementary Figure 7. The secondary structures of ATXN2L mRNA predicted by RNAfold and its free energy

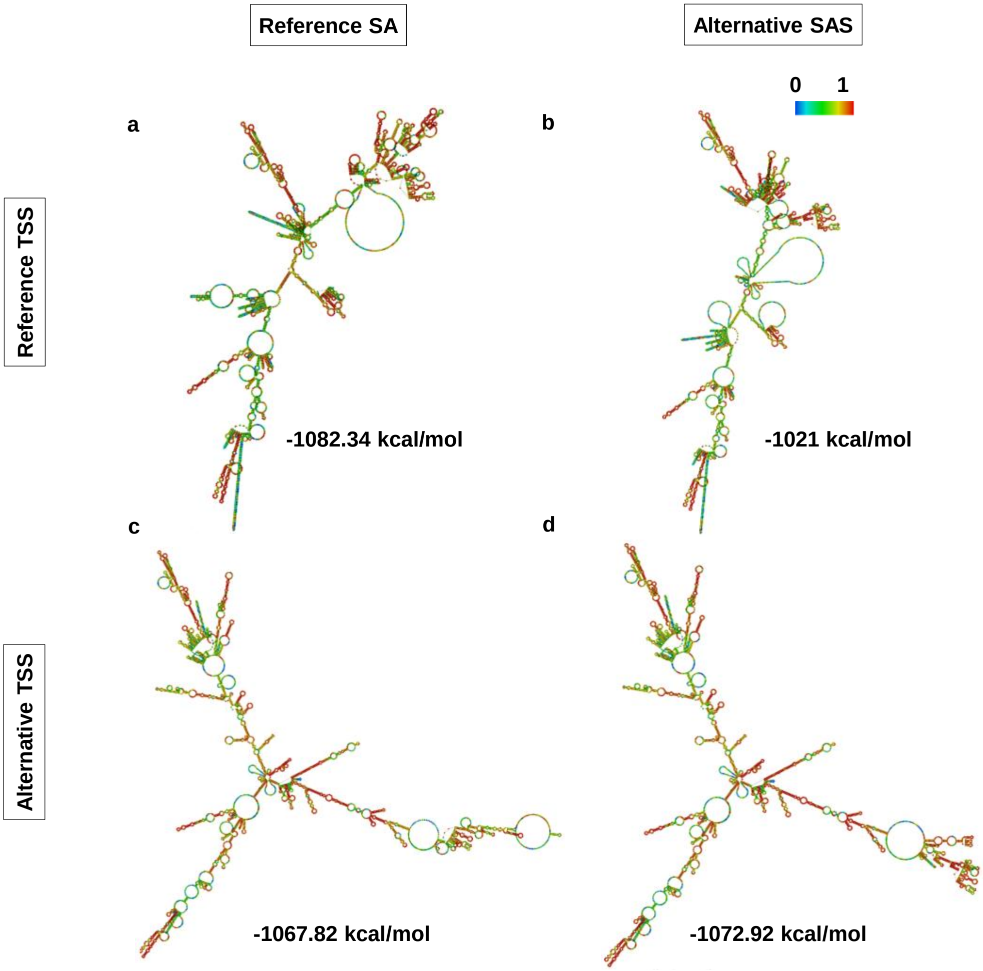

a. The predicted mRNA structure of ATXN2L isoform with reference TSS and reference SAS.
b. The predicted mRNA structure of ATXN2L isoform with reference TSS and alternative SAS.
c. The predicted mRNA structure of ATXN2L isoform with alternative TSS and reference SAS.
d. The predicted mRNA structure of ATXN2L isoform with alternative TSS and alternative SAS.

Supplementary Figure 8. GSEA analysis for i-rQTL isoforms of SNRPC using GEUVADIS RNA-seq dataset

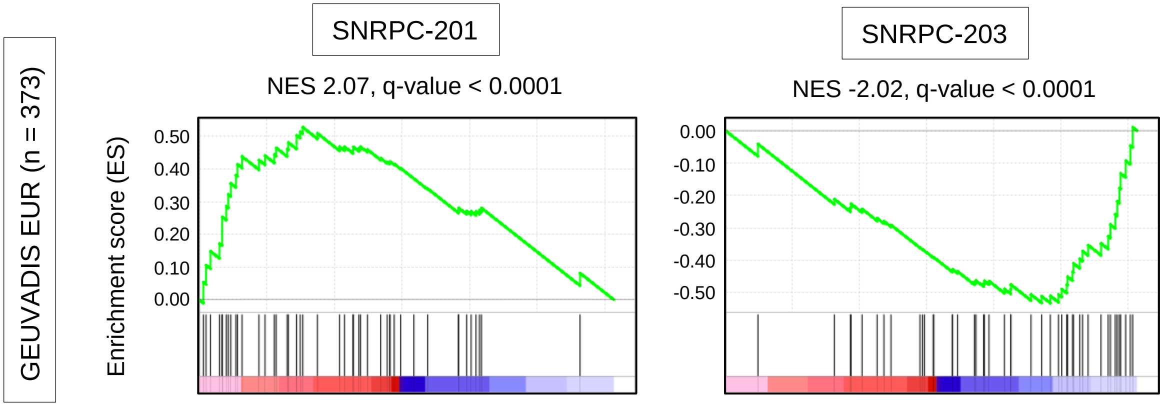

We performed the same GSEA analysis as in Figure 6g for validation, using RNA-seq data from 373 LCLs (European population) of the GEUVADIS project. Similar to the results using peripheral blood in the main text, *SNRPC* isoforms affected the interferon signature genes. (*SNRPC-201* NES 2.07, q-value < 0.0001; *SNRPC-203* NES -2.02, q-value < 0.0001).

Supplementary Figure 9. Trans-eQTL effect of sQTLs with/without protein-structure changes

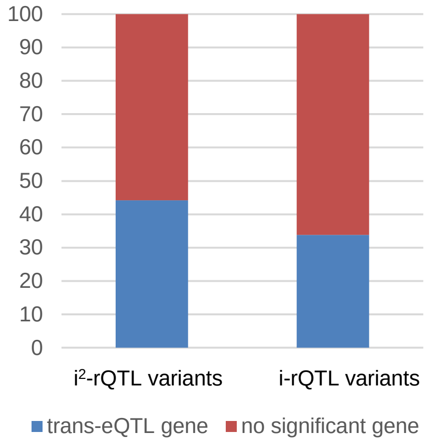

Trans-eQTL effects of sQTLs with protein-structure changes were evaluated using eQTLgen database. In comparison with i-rQTL without protein-structure changes (142 variants), i^2-rQTLs with protein-structure changes (188 variants) had a significantly higher proportion of trans-eQTL effects, as expected (44.1 % versus 33.8 %, $p = 0.0366$ in one-sided Fisher's exact test).

Supplementary Figure 10. Comparison of i^2-rQTL effects of B-cells from Japanese and LCLs from Europeans

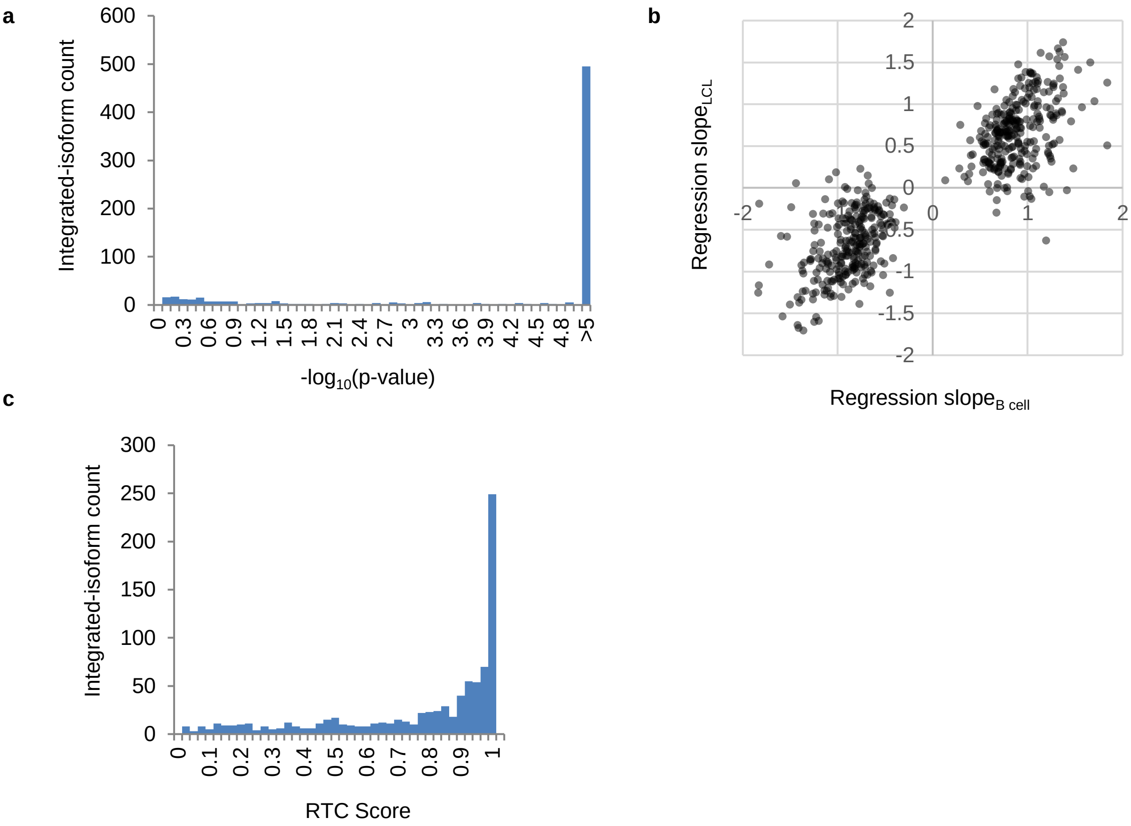

a. The distribution of -$\log_{10}$(i^2-rQTL p-values) in LCLs (GEUVADIS EUR 373 samples) for significant i^2-rQTL integrated-isoforms in B-cells.

b. Comparison of the beta values of the significant i^2-rQTL effect in both analyses. 96.4% of the directions of the i^2-rQTL effect were consistent.

c. Evaluation of the co-localization of significant i^2-rQTL effects in both analyses using RTC Score.

Supplementary Figure 11. Examples of isoforms differentially detected by i-rQTL analysis and LeafCutter.

a. *SNRPE* gene had two isoforms with different TSS, and the long isoform had all the junctions of the short isoform. The sQTL effect of this gene was identified by i-rQTL analysis but not by LeafCutter.

b. *GDAP2* gene had two isoforms with different PAS, and the long isoform had all the junction of the short isoform. The sQTL effect of this gene was identified by i-rQTL analysis but not by LeafCutter.

c. *SRSF11* gene had three isoforms. The bottom isoform had an alternative TSS, and all its junctions were shared by the other two isoforms. The latter two isoform had a mutually exclusive junction, which is focused in the box. The sQTL effect of this gene was identified by both i-rQTL analysis and LeafCutter.

Supplementary Figure 11. Examples of isoforms differentially detected by i-rQTL analysis and LeafCutter.

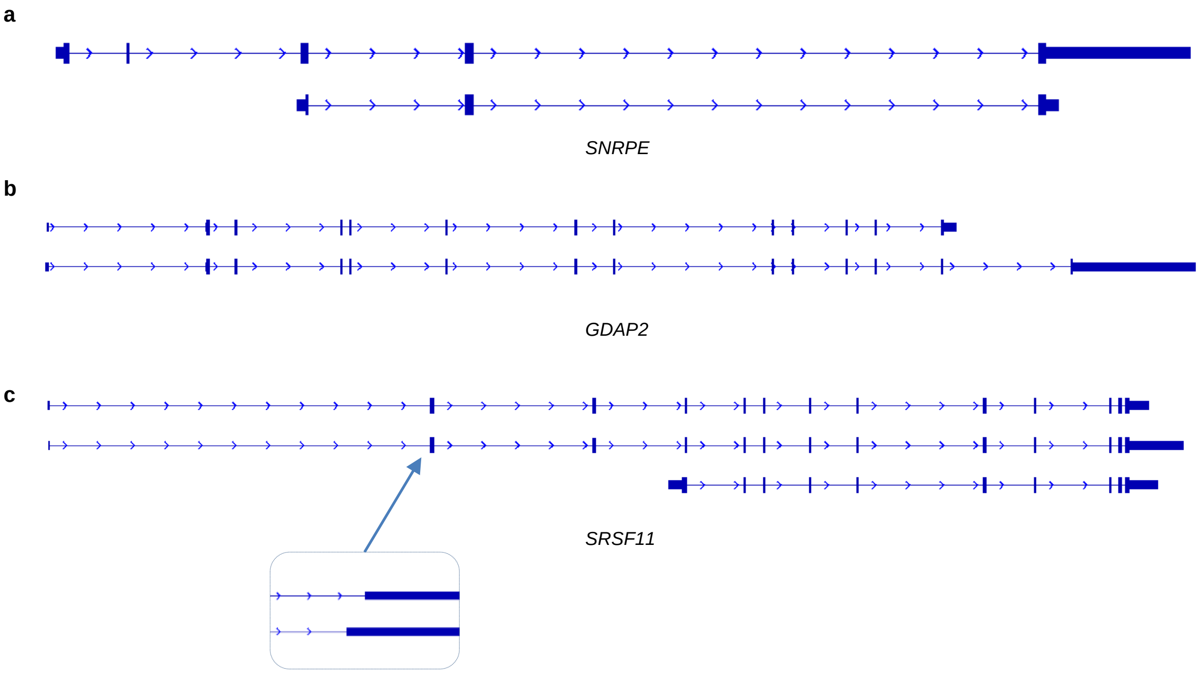

a. *SNRPE* gene had two isoforms with different TSS, and the long isoform had all the junctions of the short isoform. The sQTL effect of this gene was identified by i-rQTL analysis but not by LeafCutter.
b. *GDAP2* gene had two isoforms with different PAS, and the long isoform had all the junction of the short isoform. The sQTL effect of this gene was identified by i-rQTL analysis but not by LeafCutter.
c. *SRSF11* gene had three isoforms. The bottom isoform had an alternative TSS, and all its junctions were shared by the other two isoforms. The latter two isoform had a mutually exclusive junction, which is focused in the box. The sQTL effect of this gene was identified by both i-rQTL analysis and LeafCutter.

Supplementary Table 1. Significant QTL identified in each analysis

QTL type		B	$CD4^+T$	$CD8^+T$	Mono	NK
gene eQTL	Genes	2,840	3,226	2,476	3,889	2,829
i-eQTL	Isoforms	4,159	4,591	3,707	6,131	4,292
	Genes	2,584	2,905	2,279	3,662	2,683
i-rQTL	Isoforms	1,699	1,872	1,512	2,537	1,816
	Genes	974	1,118	917	1,438	1,088

Supplementary Table 2. Significant i^2-eQTL/i^2-rQTL identified in each analysis

QTL type		B	$CD4^+T$	$CD8^+T$	Mono	NK
i^2-eQTL	Isoforms	3,217	3,594	2,855	4,722	3,247
	Genes	2,633	2,922	2,319	3,716	2,653
i^2-rQTL	Isoforms	694	818	694	1,052	765
	Genes	416	488	425	615	449

Supplementary Table 3. Significant QTLs co-localized with GWAS effects

QTL type		B	$CD4^+T$	$CD8^+T$	Mono	NK
i-eQTL	Isoforms	831	908	705	1,283	827
i^2-eQTL	Isoforms	779	878	674	1,197	820
i-rQTL	Isoforms	294	306	242	406	295
i^2-rQTL	Isoforms	203	227	185	307	212

Supplementary Table 4. Significant i-rQTL on CDS incomplete isoforms

Isoform type		B	$CD4^+T$	$CD8^+T$	Mono	NK
i-rQTL	Isoforms	219	256	175	337	224
co-localized with GWAS	isoforms	83	81	59	129	71

Supplementary Table 5. 37 CDS incomplete isoforms analyzed by long-read capture RNA-seq

gene name	CDSI id	CDSI type	CDS completion	unique peptides	PeptideAtlas	unique junction	cell types	Representative GWAS trait
ACTN4	ENST00000586538.1	5' and 3'-CDSI	Yes	Yes	Yes	19:38709476:38711276	M/P	Heart rate
AP000295.1	ENST00000433395.6	5' and 3'-CDSI	Yes	Yes	No	21:33252830:33268394	N	Blood protein levels
ATP11A	ENST00000415301.1	5'-CDSI	No	Yes	Yes	13:112875941:112880546	M	Idiopathic pulmonary fibrosis
ATXN2L	ENST00000566946.5	5'-CDSI	Yes	Yes	No	16:28835399:28835546	B/4/8/M/N/P	Intelligence
B4GALT7	ENST00000507061.1	5'-CDSI	Yes	No	No	5:177604541:177605045	M/P	Methadone dose in opioid dependence
BST1	ENST00000514989.1	5'-CDSI	Yes	Yes	No	4:15722934:15737787	M/P	Parkinson's disease
CARD9	ENST00000641290.1	3'-CDSI	Yes	No	No	9:136371970:136373532	M	Inflammatory bowel disease
CEP250	ENST00000425096.1	5' and 3'-CDSI	Yes	Yes	No	20:35474052:35474794	4	Height
CHPF2	ENST00000465601.1	5'-CDSI	Yes	Yes	No	7:151236590:151236956	8	Height
DPP7	ENST00000473532.5	5'-CDSI	Yes	Yes	No	9:137111754:137111941	B/4/8/M/N/P	Cerebrospinal fluid biomarker levels
EXTL3	ENST00000521473.5	5' and 3'-CDSI	No	Yes	No	8:28737663:28750657	M/N/P	White matter microstructure
FANCA	ENST00000563318.1	5'-CDSI	Yes	Yes	No	16:89762793:89764890	M/N/P	Low tan response
FCGRT	ENST00000599701.5	3'-CDSI	Yes	No	No	19:49512750:49513882	M	Triglyceride levels
FECH	ENST00000592699.5	3'-CDSI	Yes	Yes	No	18:57554952:57562874	4	Lobe attachment
HLA-DPB1	ENST00000416804.1	5'-CDSI	No	Yes	No	6:33085906:33086219	B/8	ANCA-associated vasculitis
HLA-DQA1	ENST00000496318.5	3'-CDSI	No	Yes	No	6:32642253:32642952	B/M/N/P	Childhood steroid-sensitive nephrotic syndrome
HSP90B1	ENST00000550595.1	5'-CDSI	Yes	Yes	Yes	12:103947430:103953386	B/4/8/M/N/P	Blood protein levels
IFITM2	ENST00000527146.1	5'-CDSI	No	Yes	No	11:308889:309013	N	Monocyte percentage of white cells
IGHMBP2	ENST00000539224.2	5'-CDSI	No	Yes	No	11:68914350:68914823	B/4/M/N/P	High density lipoprotein cholesterol levels
LILRA2	ENST00000472992.1	5'-CDSI	Yes	Yes	No	19:54577723:54587201	M/P	Blood protein levels
LY9	ENST00000368035.1	5'-CDSI	Yes	Yes	No	1:160823796:160827748	B/4/8/N/P	Lymphocyte counts
MS4A7	ENST00000530027.5	3'-CDSI	Yes	Yes	No	11:60383288:60389390	M	Heel bone mineral density
NCAPG2	ENST00000441982.5	5'-CDSI	Yes	Yes	No	7:158646563:158652293	P	Testicular germ cell tumor
NDUFAF7	ENST00000441905.1	5'-CDSI	Yes	Yes	No	2:37248426:37253086	B/4/8/M/P	Schizophrenia
NFYC	ENST00000372669.8	3'-CDSI	Yes	Yes	No	1:40763046:40763363	M	Platelet count
OAS1	ENST00000553152.1	5'-CDSI	Yes	Yes	No	12:112917700:112918597	4/8/P	Chronic lymphocytic leukemia
PACS1	ENST00000524784.1	5'-CDSI	No	Yes	No	11:66216775:66217506	B/4	Bipolar disorder
PCMTD1	ENST00000519554.5	5'-CDSI	Yes	Yes	No	8:51827361:51831444	B/M	Major depressive disorder
PROCR	ENST00000634509.1	5'-CDSI	Yes	Yes	Yes	20:35176446:35215893	N	Estimated glomerular filtration rate
QPCT	ENST00000404976.5	3'-CDSI	Yes	Yes	No	2:37344851:37359580	M/P	Blood protein levels
RAB2A	ENST00000466595.5	5'-CDSI	Yes	Yes	No	8:60576297:60584208	B	Hypothyroidism
SH3TC1	ENST00000507891.1	5'-CDSI	Yes	Yes	No	4:8214580:8215172	P	Glaucoma
STAU1	ENST00000456866.2	3'-CDSI	Yes	Yes	No	20:49159146:49174195	M	Schizophrenia
TMTC3	ENST00000551088.1	3'-CDSI	No	Yes	No	12:88148504:88154288	4	Sunburns
TNNT1	ENST00000587089.6	5'-CDSI	Yes	Yes	No	19:55142057:55145544	Mo	Height
TP53I13	ENST00000577934.5	5'-CDSI	Yes	Yes	No	17:29571719:29571832	P	Height
TPGS2	ENST00000590652.5	5'-CDSI	Yes	Yes	No	18:36780188:36781799	B/N	QRS duration

B; B cell, 4; $CD4^+$ T cell, 8; $CD8^+$ T cell, M; Monocyte, N; NK cell, P; PBL
