# Supplementary material for: Splicing QTL analysis focusing on coding sequences reveals mechanisms for disease susceptibility loci
Source: Nat Commun. 2022 Aug 24;13:4659. doi: 10.1038/s41467-022-32358-1 (PMC9402578; doi:10.1038/s41467-022-32358-1)
Supplement: Supplementary file 3 — Description of Additional Supplementary Files [file 41467_2022_32358_MOESM3_ESM.pdf]

Description of Additional Supplementary Files

File Name: Supplementary Data 1
Description: Significant i^2-rQTLs (FDR $\leq$ 0.05)

File Name: Supplementary Data 2
Description: i^2-rQTLs co-localized with GWAS traits

File Name: Supplementary Data 3
Description: Translation sequences of integrated-isoforms in i^2-rQTL analysis (RTC ≥ 0.8, r^2 ≥ 0.8)

File Name: Supplementary Data 4
Description: Significant i-rQTLs for CDS incomplete isoforms (FDR $\leq$ 0.05)

File Name: Supplementary Data 5
Description: i-rQTLs for CDS incomplete isoforms co-localized with GWAS traits (RTC ≥ 0.8, r^2 ≥ 0.8)

File Name: Supplementary Data 6
Description: GTF file of completed CDS incomplete isoforms

File Name: Supplementary Data 7
Description: Sequences of the capture probe set for CDS incomplete isoforms
